# Two novel murine esophageal cancer cell line models exhibiting distinct sensitivities to immunotherapy

**DOI:** 10.1016/j.gendis.2026.102059

**Published:** 2026-01-30

**Authors:** Jinling Zhang, Yichen Yao, Jiajia Hu, Chenyi Wu, Jiaying Chen, Yanxing Chen, Zixin Qin, Qi Zhao, Runjie Huang, Feng Wang, Yingnan Wang

**Affiliations:** a Department of Medical Oncology, Sun Yat-sen University Cancer Center, State Key Laboratory of Oncology in South China, Guangdong Provincial Clinical Research Center for Cancer, Sun Yat-sen University, Guangzhou, Guangdong 510000, China; b Research Unit of Precision Diagnosis and Treatment for Gastrointestinal Cancer, Chinese Academy of Medical Sciences, Guangzhou, Guangdong 510000, China; c State Key Laboratory of Oncology in South China, Sun Yat-sen University Cancer Center, Guangzhou, Guangdong 510000, China; d Department of Anesthesiology, Sun Yat-sen University Cancer Center, Guangzhou, Guangdong 510000, China

Immunotherapy has emerged as a frontline treatment for advanced esophageal squamous cell carcinoma (ESCC). Still, therapeutic resistance remains a major challenge.^1^ The development of robust preclinical models is critical for ESCC, as current murine-derived cell lines are limited to AKR^2^ and mEC25 ^3^, which are scarce compared with human-derived counterparts. In this study, we established two primary ESCC cell lines, SYEC2 and SYEC21, derived from orthotopic tumors in C57BL/6NJ mice induced by the carcinogen 4-Nitroquinoline 1-oxide (4-NQO). Histopathological analysis confirmed their squamous cell carcinoma characteristics. Both cell lines exhibited robust proliferative and migratory capacities *in vitro* and demonstrated differential sensitivity to chemotherapeutic agents (5-fluorouracil, paclitaxel, cisplatin, and irinotecan). *In vivo*, subcutaneous injection of SYEC2 and SYEC21 can form tumors, with SYEC2 showing superior responsiveness to immunotherapy. Mechanistically, SYEC2 tumors displayed higher CD8^+^ T cell infiltration, lower CD4^+^ T cell presence, reduced major histocompatibility class I (MHC-I) expression, and elevated programmed death ligand-1 (PD-L1) levels compared with SYEC21. Whole-genome and transcriptome analyses further revealed substantial genetic heterogeneity between the two models. Collectively, we present two immunologically distinct murine ESCC models that recapitulate key features of human disease and facilitate the investigation of mechanisms underlying immunotherapy resistance.

To develop a syngeneic mouse model, we administered 4-NQO in the drinking water of C57BL/6NJ mice as described in Figure S1A,^3^ resulting in esophageal tumorigenesis (Fig. S1B). Pathological analysis confirmed the malignant phenotype in primary tumors (Fig. S1C). We subsequently performed subcutaneous transplantation into immunocompetent C57BL/6NJ mice and identified two tumors (No. 2 and No. 21) with robust growth (Fig. 1A and B). Both tumors retained histopathological features of the original specimens (Fig. S1D), exhibiting strong expression of squamous markers (pan-cytokeratin, CK5/6, p40, and p63), alongside low levels of E-cadherin. Notably, tumor No. 2 showed higher PD-L1 expression compared with No. 21 (Fig. 1C).

**Figure 1:**
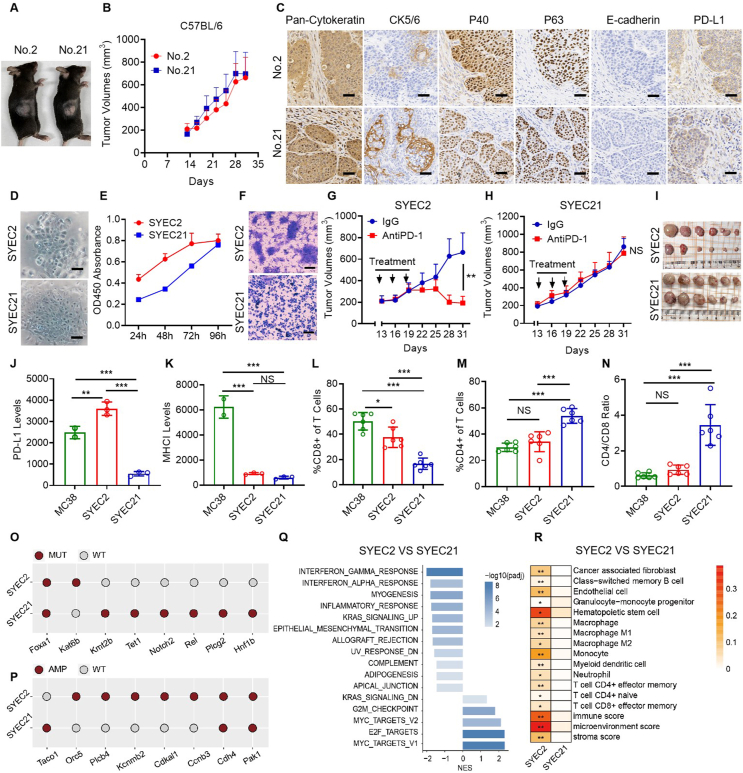
Induction and optimization of two primary esophageal cancer cell lines, SYEC2 and SYEC21, which were derived from orthotopic tumors in C57BL/6NJ mice. **(A, B)** Tumor tissues (No. 2 and No. 21) exhibiting enhanced proliferation and growth abilities after subcutaneous transplantation. **(C)** Immunohistochemical staining of Pan-cytokeratin, CK5/6, p63, p40, E-cadherin, and PD-L1 in xenograft tumors derived from No. 2 and No. 21 (scale bar: 50 μm). **(D)** Morphological images of 2D cell lines (scale bar: 50 μm). **(E)** Cell viability assay results for SYEC2 and SYEC21 cells at the 20th generation. **(F)** Representative images of Transwell migration assays for SYEC2 and SYEC21 cells (scale bar: 50 μm). **(G, H)** Tumor growth curve for SYEC2 (G) and SYEC21 (H) (*n* = 6 per group) following PD-1 antibody treatment compared with IgG control. 100 μg/mouse of PD-1 antibody or isotype IgG was intraperitoneally injected on days 13, 16, and 19. **(I)** The images of SYEC2 and SYEC21 tumors at the experimental endpoint. **(J, K)** The PD-L1 (J) and MHC-1I (K) levels in MC38, SYEC2, and SYEC21 cells (*n* = 3 per group) were obtained using flow cytometry. **(L, M)** Percentages of CD8^+^ (L) and CD4^+^ T (M) cells in MC38, SYEC2, and SYEC21 tumors. **(N)** CD4^+^/CD8^+^ T cell ratio in MC38, SYEC2, and SYEC21 tumors. **(O)** Annotated mutant genes identified in both models. **(P)** Genes with high amplification observed in both models. **(Q)** Gene set enrichment analysis comparing SYEC21 with SYEC2. NES > 0 indicates enrichment in SYEC21, while NES < 0 indicates enrichment in SYEC2. **(R)** Comparison of immune cell infiltration between SYEC2 and SYEC21. Differences between groups were evaluated using two-way ANOVA (G, H) and one-way ANOVA (J–N). Statistical significance levels were denoted as follows. ∗*P* < 0.05, ∗∗*P* < 0.01, and ∗∗∗*P* < 0.001; NS, not significant.

To develop a model replicating original tissue histology and lineage hierarchy *in vitro*, 3D organoids were generated from single-cell suspensions of orthotopic tumors No. 2 and No. 21, which were designated SYEC2 and SYEC21. Both organoids proliferated efficiently *in vitro*, with morphology validated by hematoxylin-eosin staining (Fig. S1E). After five passages *in vivo*, a two-step digestion method was employed to selectively isolate tumor cells while removing fibroblasts. Subsequent subculturing in 2D revealed distinct morphological differences between SYEC2 and SYEC21 (Fig. 1D). These cells maintained consistent growth over 20 generations at a split ratio of 1:3 every three days, demonstrating robust proliferation capabilities, which were also confirmed through cell proliferation and migration assays (Fig. 1E and F). Drug sensitivity assays revealed differential responses to ESCC therapies, including 5-fluorouracil, paclitaxel, cisplatin, and irinotecan (Fig. S2A–S2D). For immunotherapy response assessment, cells were inoculated subcutaneously into C57BL/6NJ mice and treated with programmed cell death protein 1 (PD-1) antibody upon tumor growth to 200 mm^3^. Both cell lines demonstrated robust tumor formation *in vivo*, with SYEC2 responding more effectively to PD-1 blockade (Fig. 1G–I; Fig. S2E–S2G).

PD-L1 is a critical immune checkpoint regulator that drives T cell exhaustion and modulates anti-tumor immune responses.^4^ MHC-I molecules are crucial for antigen presentation, facilitating the recognition of tumor-associated antigens by CD8^+^ T cells via T cell receptors.^5^ To characterize the expression of PD-L1 and MHC-I in SYEC2 and SYEC21 cells, we performed flow cytometry analysis using these cell lines alongside MC38 cells, which serve as a comparison due to their classification as a “hot” tumor model. Our results demonstrated that PD-L1 expression levels were significantly higher in MC38 and SYEC2 cells compared with SYEC21 (Fig. 1J; Fig. S3A). Conversely, MHC-I expression levels were notably lower in both SYEC2 and SYEC21 cells relative to MC38 cells (Fig. 1K; Fig. S3B). The elevated PD-L1 expression observed in SYEC2 might explain its better response to PD-1 antibody treatment *in vivo*. Additionally, flow cytometry revealed significant CD8^+^ T cell infiltration in both MC38 and SYEC2 tumors, compared with SYEC21 (Fig. 1L; Fig. S3C–S3E). In contrast, CD4^+^ T cell infiltration was significantly lower in MC38 and SYEC2 tumors than in SYEC21 (Fig. 1M), resulting in a significantly higher CD4^+^/CD8^+^ ratio in SYEC21 (Fig. 1N). These findings suggest that distinct patterns of T cell infiltration may contribute to the differing immunotherapy responses between SYEC2 and SYEC21.

We subsequently conducted comprehensive whole-genome sequencing and used the C57BL/6NJ mouse genome as a reference, identifying somatic mutations in both SYEC2 and SYEC21 models. As expected, missense mutations were predominant, with SYEC21 displaying a significantly higher number of nonsynonymous mutations than SYEC2, reflecting the random mutational landscape typical of carcinogen-induced tumors (Fig. S4A). Notably, mutations in Foxa1 were identified in both models; Kat6b was mutated in SYEC2, and Kmt2b, Tet1, and Notch2 were mutated in SYEC21 (Fig. 1O). Analysis of somatic copy number alterations (SCNAs) revealed that SYEC2 had a slightly higher SCNA burden than SYEC21 (Fig. S4B and S4C). Both models exhibited significant amplification of Pak1 and Cdh4, with additional specific amplifications identified in SYEC2 (Ccnb3, Cdkal1, Kcnmb2, Plcb4, Orc5) and only Taco1 amplified in SYEC21 (Fig. 1P). These findings suggest that SYEC21 may be primarily driven by mutations, indicating a complex genomic landscape, whereas SYEC2 appears to be more prominently influenced by SCNAs.

To characterize the transcriptomic features of the ESCC mouse models, we conducted transcriptome sequencing (Fig. S5A). Our analysis revealed that pathways associated with immune and inflammatory responses, including interleukin-2 (IL-2)–signal transducer and activator of transcription 5 (STAT5) signaling, tumor necrosis factor alpha (TNFα)–nuclear factor kappa B (NFκB) signaling, and interferon alpha (IFN-α) and interferon gamma (IFN-γ) responses, were significantly enriched in SYEC2 tumor samples than normal esophageal tissues (Fig. S5B). In contrast, pathways associated with cell proliferation, including E2F transcription factor targets, G2M checkpoint, and MYC transcription factor targets, were notably enriched in SYEC21 (Fig. S5C), highlighting its malignant and highly proliferative nature. Furthermore, the IFN-α and IFN-γ response pathways were more activated in SYEC2 than in SYEC21 (Fig. 1Q), indicating a robust immune response of SYEC2 that may underpin its distinct efficacy against anti-PD-1 treatment. To dissect immune heterogeneity between **SYEC2** (immunotherapy-sensitive) and **SYEC21** (therapy-resistant) models, we employed the landscape of immune cell heterogeneity using xCell 1.0 (a computational tool quantifying 64 immune/stromal cell types). A heatmap indicated varying levels of infiltration among immune cell types, including myeloid dendritic cells, CD4^+^ effector memory T cells, and CD8^+^ effector memory T cells. Notably, SYEC2 exhibited higher recruitment levels of these immune cells and an elevated immune score, suggesting a potentially stronger anti-tumor immune response compared with SYEC21 (Fig. 1R; Fig. S5C). In summary, SYEC2 demonstrated enhanced immune cell infiltration and cytotoxicity within the tumor microenvironment.

In conclusion, we established and characterized two novel esophageal cancer cell lines, SYEC2 and SYEC21, focusing on histopathological features, drug sensitivity, and multi-omics analyses. Both models demonstrate promising applicability in research; notably, SYEC2 demonstrated superior sensitivity to anti-PD-1 therapy compared with SYEC21. These models will promote preclinical ESCC research by offering genetically defined, immunologically distinct tools to accelerate the development of precision therapies for heterogeneous ESCC subtypes.

## CRediT authorship contribution statement

**Jinling Zhang:** Writing – review & editing, Writing – original draft, Resources, Data curation, Conceptualization. **Yichen Yao:** Writing – review & editing, Software, Formal analysis, Data curation. **Jiajia Hu:** Writing – review & editing, Validation, Investigation. **Chenyi Wu:** Writing – review & editing, Validation, Investigation. **Jiaying Chen:** Writing – review & editing, Software, Formal analysis, Data curation. **Yanxing Chen:** Software, Formal analysis. **Zixin Qin:** Software, Formal analysis. **Qi Zhao:** Validation, Investigation. **Runjie Huang:** Software, Formal analysis. **Feng Wang:** Writing – review & editing, Project administration, Funding acquisition, Conceptualization. **Yingnan Wang:** Writing – review & editing, Supervision, Project administration, Funding acquisition.

## Ethics declaration

The study was approved by the Ethics Committee of the Institutional Animal Care and Use Committee of Sun Yat-sen University Cancer Center (L025504202107035).

## Data availability

The datasets used and/or analyzed during the current study are available from the corresponding authors upon reasonable request.

## Funding

This work has been supported by the National Natural Science Foundation of China (No. 82425048 to F.W.), the New Cornerstone Science Foundation (China) (No. XPLORER PRIZE to F.W.), the Beijing Xisike Clinical Oncology Research Foundation (China) (No. Y-QL202202-0089 to F.W.), Guangdong Esophageal Cancer Institute Science and Technology Program (China) (No. Q202005 to Y.N.W.), and the Young Talents Program of Sun Yat-sen University Cancer Center (Guangdong, China) (No. YTP-SYSUCC-0018 to F.W.).

## Conflict of interests

The authors declared no conflict of interests.
