# Supplementary material for: Two novel murine esophageal cancer cell line models exhibiting distinct sensitivities to immunotherapy
Source: Genes Dis. 2026 Jan 30;13(5):102059. doi: 10.1016/j.gendis.2026.102059 (PMC13153458; doi:10.1016/j.gendis.2026.102059)
Supplement: Multimedia component 1 [file mmc1.docx]

**Supplementary figures**

Supplementary figure 1

**
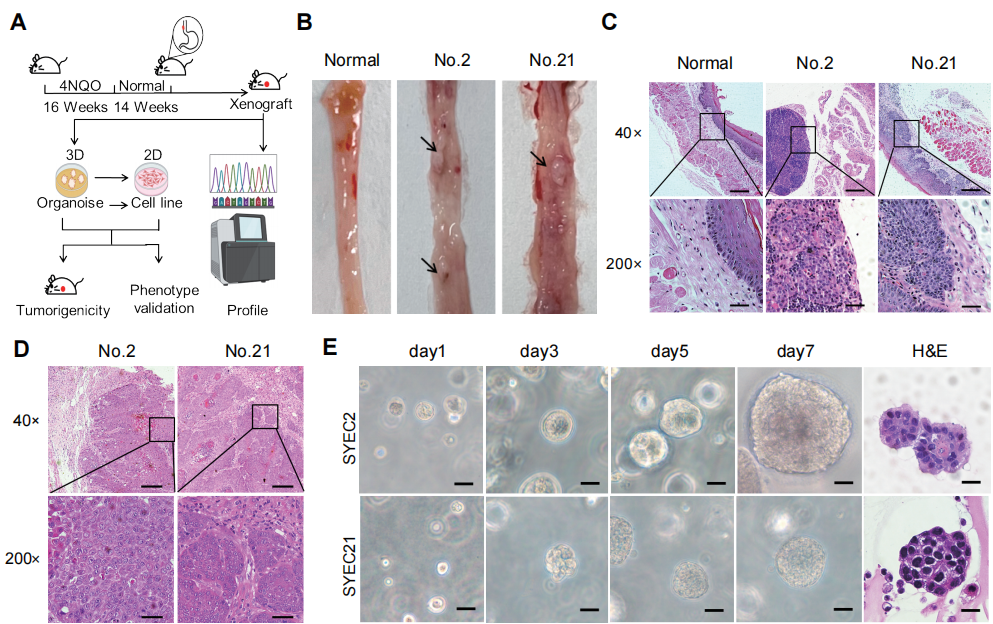
**

Fig. S1. Induction and optimization of syngeneic mouse models for esophageal cancer. **(A)** Procedure for establishing and verifying syngeneic mouse models. **(B)** Isolated esophageal tissues from the control and experimental groups were examined after 30 weeks. Normal tissue was from the control group receiving normal water, while No. 2 and No. 21 were from the experimental group receiving 4NQO for 16 weeks and then regular water for 14 weeks. **(C)** H&E staining of normal, No. 2, and No. 21 esophageal tissues (scale bar: 50 μm). **(D)** H&E staining of the selected transplanted tumor tissues (scale bar: 50 μm). **(E)** Brightfield morphology of organoids cultured in 3D conditions on days 1,3,5,7, and morphology from H&E staining of 3D organoids.

Supplementary figure 2

**
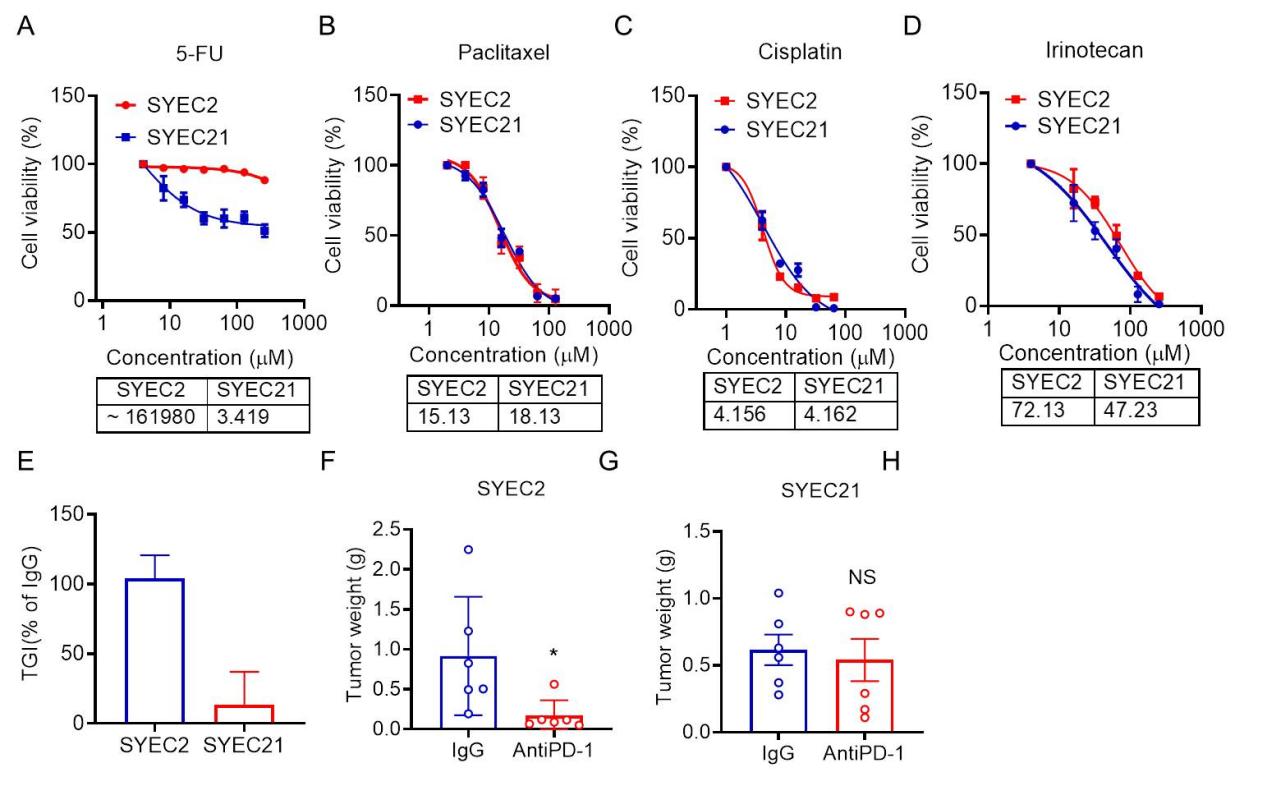
**Fig. S2. Divergent therapy response to chemotherapeutics and PD-1 antibody between SYEC2 and SYEC21. **(A–D)** Cell viability assay after treatment with varying concentrations of 5-FU, Paclitaxel, Cisplatin, and Irinotecan. IC50 analysis was performed after 72 hours of treatment. **(A)** SYEC2 exhibited resistance to 5-FU, while SYEC21 showed higher sensitivity with an IC50 of 3.4 μM. (B) IC50 values for Paclitaxel were 15.1 μM for SYEC2 and 18.1 μM for SYEC21. **(C)** Both SYEC2 and SYEC21 had an IC50 of 4.1 μM for Cisplatin. **(D)** For Irinotecan, SYEC2 had an IC50 of 72.1 μM, whereas SYEC21 showed a lower IC50 of 47.2 μM. **(E)** A summary of tumor growth inhibition (TGI) of SYEC2 and SYEC21 tumors on day 31 after treatment. **(F)** Weight of SYEC2 tumor at the experimental endpoint. **(H)** Weight of SYEC21 tumor at the experimental endpoint. Differences between groups were evaluated using Student's t-test (F and G). Statistical significance levels were denoted as follows: *, *P* < 0.05, NS, not significant.

Supplementary figure 3


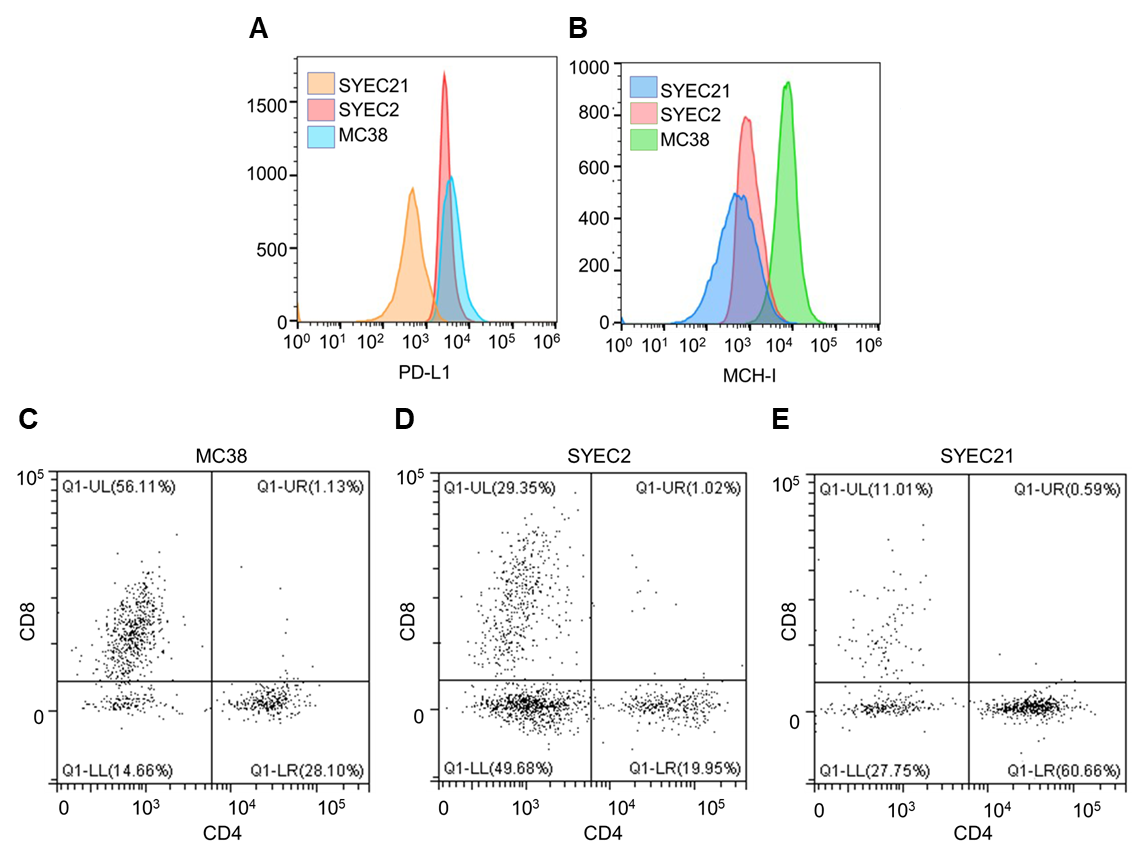


Fig. S3. Distinct PD-L1, MHC-I expression and immune microenvironments among MC38, SYEC2 and SYEC21. The PD-L1 **(A)** and MHC-I **(B)** levels of MC38, SYEC2, and SYEC21 cells (n = 2-3 per group). **(C-E)** Representative image of flow cytometry gating from MC38, SYEC2 and SYEC21 tumors.

Supplementary figure 4


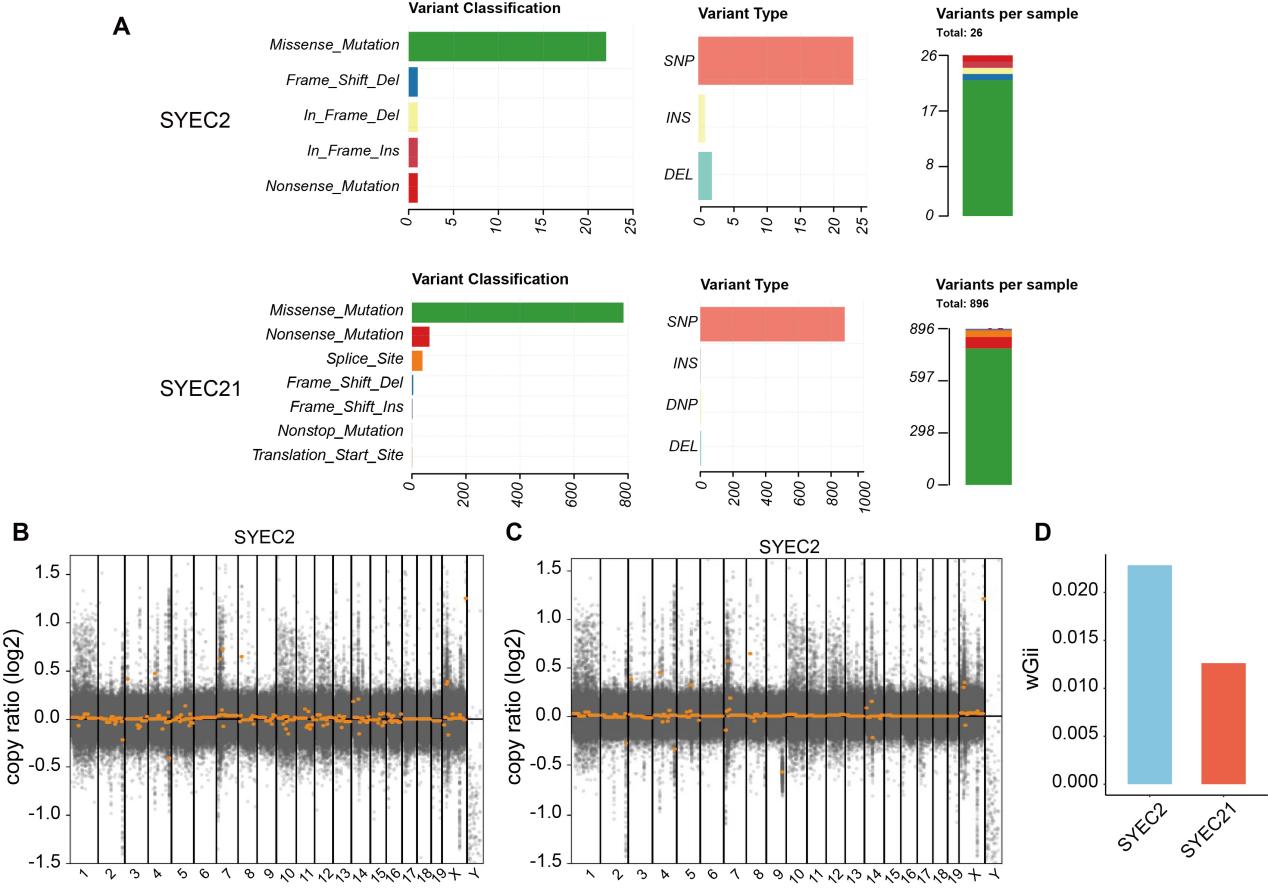


Fig. S4. Genomic profile of SYEC2 and SYEC21. **(A)** Overview of somatic mutations in both models. **(B-D)** Somatic copy number alteration (SCNA) burden of the two models.

Supplementary figure 5


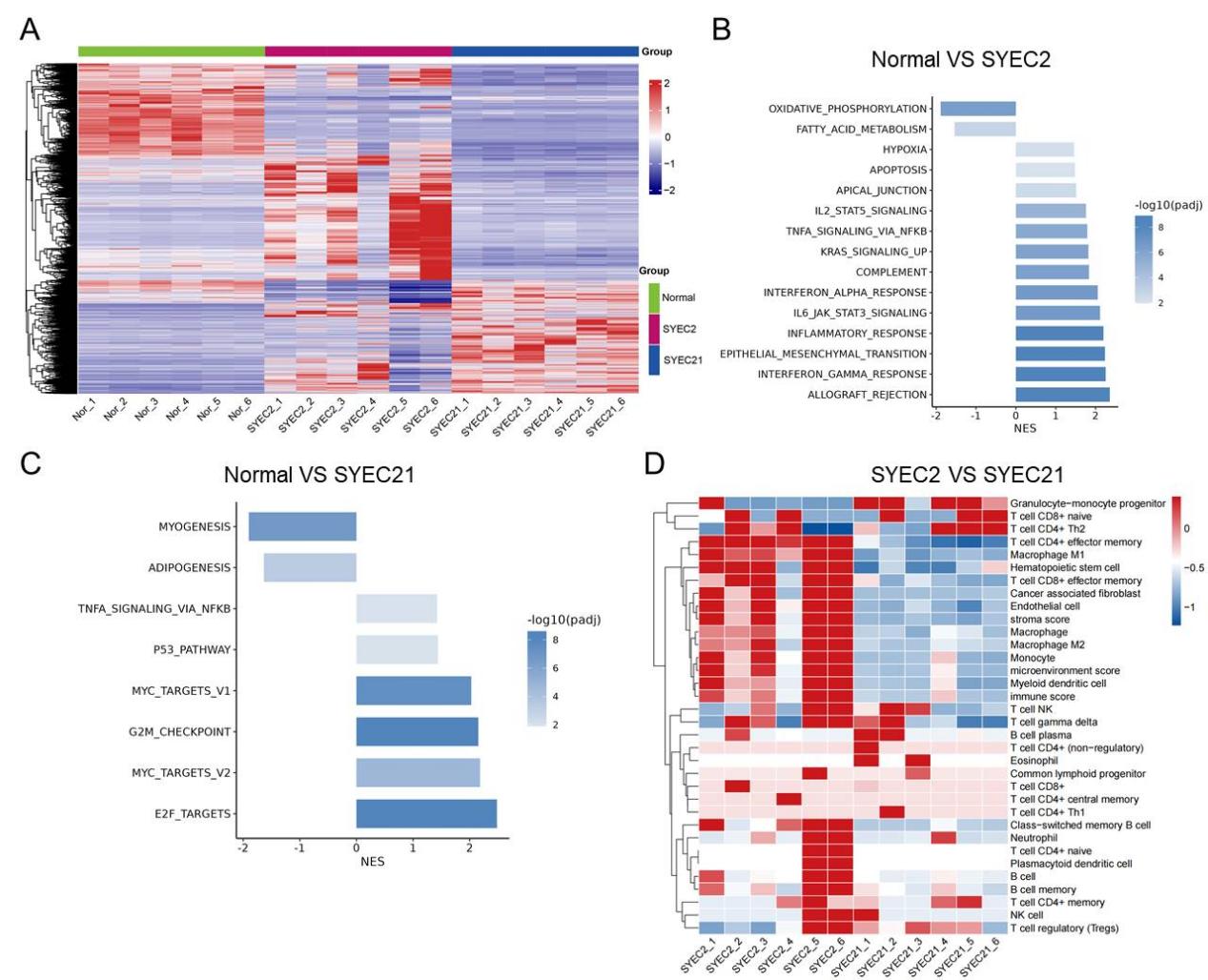


Fig. S5. Transcriptome landscape of SYEC2 and SYEC21 compared to normal esophagus. **(A)** Heatmap depicts differentially expressed genes among normal tissues, SYEC2, and SYEC21 tumors. **(B-C)** Gene set enrichment analysis comparing SYEC2 with normal esophagus and SYEC21 with normal esophagus. The X-axis represents the normalized enrichment score, the y-axis lists gene set names collected by MSigDB, and the color shades indicate statistical significance. NES > 0 indicates enrichment in SYEC2, while NES < 0 indicates enrichment in the normal esophagus. **(D)** Heatmap depicts the differences in the composition of various cell types between two sample groups, SYEC2 and SYEC21.
