# Supplementary material for: Two novel murine esophageal cancer cell line models exhibiting distinct sensitivities to immunotherapy
Source: Genes Dis. 2026 Jan 30;13(5):102059. doi: 10.1016/j.gendis.2026.102059 (PMC13153458; doi:10.1016/j.gendis.2026.102059)
Supplement: Multimedia component 2 [file mmc2.docx]

**Supplementary Material and Methods**

**Carcinogen-induced Spontaneous ESCC tumor model**

Female C57BL/6 mice (5-6 weeks old, 15-18 g) were procured from Guangdong Gem Pharma Tech Co., Ltd. Animal experiments were approved by the Institutional Animal Care and Use Committee of Sun Yat-sen University Cancer Center (L025504202107035). To induce esophageal tumors in situ, mice were administered sterile drinking water supplemented with 100 μg/mL of 4-nitroquinoline-1-oxide for 16 weeks, followed by regular water for 14 weeks. At the end of the experimental period (30 weeks), all mice were euthanized, and their esophageal tissues were dissected to assess tumor formation. Tumor specimens were bisected upon collection: one half was fixed in 4% formalin and embedded in paraffin for histopathological examination to confirm the pathological type, while the other half was prepared for subsequent allogeneic transplantation.

**Transplantation of ESCC tissue**

Isolated primary esophageal tumor tissues were promptly rinsed with 1× PBS upon collection. Recipient mice, 6-week-old female C57BL/6 mice, were anesthetized with 1% sodium pentobarbital (Sigma-Aldrich, Shanghai, China). Tumor fragments were then subcutaneously implanted on the right dorsal flank of each mouse. After five weeks, subcutaneous tumors were excised and subsequently transplanted into another group of C57BL/6 mice using the same procedure. Tumors demonstrating robust growth capability were selected through repeated transplantation, exceeding ten passages to enhance tumor formation proficiency.

**Histopathological analysis**

The 4-NQO-induced primary mouse ESCC tumors, alongside No.2 and No.21 xenograft tumors, were excised and fixed in 4% formalin overnight. Following fixation, specimens underwent dehydration and were subsequently embedded in paraffin. Serial sections of the paraffin-embedded tumor tissues, each 5 μm thick, were prepared and stained with hematoxylin and eosin (H&E). Histopathological analysis was conducted by a skilled pathologist.

**Immunohistochemistry and Imaging**

The tumor tissues were dehydrated, embedded in paraffin, and sectioned into 5 µm-thick slices, followed by fixation with 4% paraformaldehyde (PFA). According to standard protocols, Immunohistochemistry (IHC) was performed on the tissue sections. Slides were stained using the following antibodies: Pan-cytokeratin (Cell Signaling Technology, CST, 4545T), E-cadherin (CST, 3195T), CK 5/6 (ZS, ZA-0683), p63 (Abcam, ab164762), p40 (Abcam, ab203826), and PD-L1 (Proteintech, 66248-1-IG). Signal Stain Detection Boost IHC/HRP rabbit (#8144S, CST) or mouse (#8125S, CST) was employed for detection purposes. Stained slides were imaged using an EVOS FL Auto microscope (Life Technologies). Subsequently, all tumor sections underwent blinded review by two experienced pathologists for comprehensive analysis.

**Establishment of mouse ESCC** **Organoids**

Organoids were derived from tissue blocks showing robust growth in C57BL/6 mice. Tumor tissue was transferred to sterile forceps, washed in 1× PBS, and minced into <5 mm fragments. These fragments were enzymatically treated with HBSS containing dispase, collagenase, and antibiotic-antimycotic, followed by incubation and centrifugation. The pellet was then incubated with Collagenase Type IV, Dispase, and Y-27632, followed by trypsin-EDTA digestion with DNase I.

After filtration and treatment with Soybean trypsin inhibitor, tumor cells were centrifuged, washed in HBSS, and resuspended in basal medium. Next, 2 × 10^5^ cells were mixed with ice-cold Matrigel (Corning, Cat#356231), plated in a pre-warmed 24-well plate, and solidified at 37°C. After solidification, organoid medium was applied (DMEM/F12 (Gibco, Cat# A4192001) medium supplemented with GlutaMax (Gibco, Cat# 35050079), N2 Supplement (Gibco, Cat# 17502001), B27 Supplement (Gibco, Cat# 17502001), 1 mM N-acetylcysteine (Sigma, Cat# A0737), 50 ng/mL human epidermal growth factor (PEPROTECH, Cat# 900-M05), 10 μM Y27632 (Sellect, Cat# S6390), and Noggin (Novoprotein, Cat# CB89), 10% conditioned medium containing Wnt3a (JFKR-Organoid) and R-spondin (JFKR-Organoid). Organoid medium was refreshed every 2-3 days. For passaging, organoids were dissociated using trypsin-EDTA and DNase I, then re-embedded in cold Matrigel for continued 3D culture.

**Establishment of mouse ESCC cell lines**

The organoids were enzymatically dissociated into single cells using 0.25% trypsin-EDTA and DNase I after 5 passages. Subsequently, 1×10^6^ cells underwent centrifugation and were resuspended in RPMI1640 medium (Gibco, Cat#C11875500BT) supplemented with 10% Fetal Bovine Serum (ExCell Bio, Cat#FSP500), 100U/mL of Penicillin and 100ug/mL Streptomycin (Gibco, Cat#15070063). Culture medium was refreshed every other day over three consecutive generations. During the initial five passages, cells were detached using a two-step digestion method: a 2-minute treatment with 0.25% Trypsin (Gibco, Cat#25200072) to remove fibroblasts, followed by PBS washing, and then a 10-minute digestion with 0.25% Trypsin. Subsequent passages employed a single-step digestion method. At passage 10, stable cell lines were successfully established. Mouse Cell STR authentication was conducted by Guangzhou Cell Cook Biotech Co., Ltd.

**Cell growth assay**

SYEC2 and SYEC21 cells (5× 10^3^ cells/well) were seeded in a 96-well plate and treated with 10 μL of CellTiter-Glo Cell Viability Assay MTS solution (Promega, Cat#G3581) at 24, 48, 72, and 96 hours post-seeding. Following a 2-hour incubation, the absorbance of the cell culture supernatant was measured at 450 nm using a Biotek microplate reader.

**Migration assay**

1 × 10^5^ cells in 100 μL of serum-free RPMI 1640 medium were seeded into the upper chamber of Corning inserts (Corning, Cat#3422), while the lower chamber contained 650 μL of complete culture medium with 20% FBS. Following a 48-hour incubation period, non-migratory cells and media from the upper chamber were removed. Cells on the lower surface of the membrane were fixed with methanol and stained using a 0.1% crystal violet solution (Sigma-Aldrich, Cat#V5265).

**Cell viability assay**

4× 10^3^ Cells were seeded in 96-well plates containing 100 μL of culture medium per well. For chemotherapeutic studies, cells were treated with varying concentrations of drugs: 5-Fluorouracil (5-FU) (0, 4, 8, 16, 32, 68, 128 μM), Irinotecan (0, 32, 64, 128, 256, 512, 1024 μM), Paclitaxel (0, 4, 8, 16, 32, 68, 128 μM), and Cisplatin (0, 4, 8, 16, 32, 68, 128, 256 μM). Drug pre-dilutions were prepared in 100 μL of the respective medium, added to each well, and incubated for 48 hours. Cell viability was assessed using the CellTiter-Glo Cell Viability Assay (Promega, Cat#G3581) after 72 hours.

**In vivo tumorigenicity of cells**

For tumor formation studies, 3 × 10^6^ cells were suspended in 100 μL of PBS and injected subcutaneously into C57BL/6 mice (6 mice per group). Tumor growth was monitored over time in the mice. Tumor volume was calculated using the formula: Volume = Length × Width × Width/2.

**Anti-PD-1 therapy in syngeneic SYEC2 and SYEC21 tumor models**

3 × 10^6^ to 4 × 10^6^ SYEC2 and SYEC21 cells were suspended in 100 μL of PBS and injected subcutaneously into groups of C57BL/6 mice (6 mice per group). Mice were treated with either anti-PD-1 antibody (100 μg/mouse; Bio X cell, Lebanon, New Hampshire, USA) or IgG isotype control (100 μg/mouse; Bio X cell) every 3 days for a total of 3 injections. Tumor growth was monitored every 3 days for approximately 5 weeks. At the end of the experiment, mice were euthanized, and tumors were excised, weighed, and subjected to histological analysis. The tumor growth inhibition rate (TGI) was calculated using the following formula: TGI (%) = {1 - [(final volume - initial volume) of the treated group / (average final volume - average initial volume) of the control group]} × 100%.

**Flow cytometry**

For animal models, fresh tumor tissues from MC38, SYEC2, and SYEC21 implantation mice were processed into single-cell suspensions. Tumors were minced into 2–4 mm pieces, excluding blood, fibrous, and necrotic areas. These tissue fragments were then enzymatically dissociated using a Tumor Dissociation Kit (Miltenyi Biotec, 130-096-730) in gentleMACS C Tubes on a gentle MACS dissociator, followed by filtration through a 70 μm cell strainer (BD Biosciences) and washing with DMEM culture medium. For cell models, MC38, SYEC2, and SYEC21 cells were dissociated into single-cell suspensions using 0.25% trypsin and passed through a cell strainer.

Cell surface staining was performed using fluorescent dye-labeled antibodies against specific antigens in a blocking buffer (PBS with 2% FBS) on ice for 30 minutes. Antibodies used included PerCP/Cyanine5.5 anti-mouse CD45 (Cat#103132), FITC anti-mouse CD3ε (Cat#100306), Brilliant Violet 650 anti-mouse CD4 (Cat#100555), APC/Fire750 anti-mouse CD8a (Cat#100766), Brilliant Violet 421 anti-mouse CD274 (PD-L1, Cat#124315), PE anti-mouse H-2Kb/H-2Db (MHC-I, Cat#114608), and Zombie UV Fixable Viability Kit (Cat#423107), all sourced from BioLegend.

Flow cytometry analysis and cell sorting were conducted using a CytoFLEX flow cytometer (Beckman Coulter), and data were analyzed with FlowJo software (Treestar). Cells from subcutaneous tumors were gated based on live cells and specific markers: CD8^+^ T cells (CD45^+^CD3^+^CD8^+^), and CD4^+^ T cells (CD45^+^CD3^+^CD4^+^). Cultured cells were assessed for PD-L1 and MHC-I levels among live cells.

**Preprocessing and sequencing**

Fresh samples were obtained from normal esophageal tissues, SYEC2, and SYEC21 subcutaneous tumors for Whole Genome Sequencing (WGS) and RNA Sequencing (RNA-seq). Genomic DNA was extracted using the DNeasy Blood & Tissue Kit (Qiagen), and DNA libraries were constructed using the NEBNext DNA Library Prep Reagent Set (BioLabs, Cat#E6000). Sequencing was performed on an Illumina NovaSeq 6000 platform (Illumina, CA, USA), generating 150 bp paired-end reads at LC-Bio Technology Co., Ltd, Hangzhou, China.

Total RNA was extracted, followed by mRNA isolation using the NEBNext® Poly(A) mRNA Magnetic Isolation Module and library construction with the NEBNext® Ultra™ II mRNA Library Prep Kit for Illumina®. High-throughput transcriptome sequencing was then conducted on an Illumina NovaSeq 6000 platform.

**Genome data processing procedure**

The raw whole genome sequencing data for SYEC2 and SYEC21 were aligned to the mm10 reference genome using bwa (0.7.17-r1188). Subsequently, GATK (4.1.2.0) was employed to perform Base Quality Score Recalibration and remove duplicate reads. The bam file of the C57BL_6NJ genome data served as a reference for identifying somatic mutations and somatic copy number alterations (SCNAs) in the two models. Somatic mutations in SYEC2 and SYEC21 were called using Mutect2, referencing the C57BL_6NJ genome data, while SCNA detection utilized CNVkit.

For annotation, mutational cancer genes were sourced from OncoKB (https://www.oncokb.org/), and annotated CNA cancer genes were obtained from DriverDBv4 (http://driverdb.tms.cmu.edu.tw/). Variant Effect Predictor (VEP v102.0) was utilized for mutation annotation. Copy number gain was defined as a copy number ratio greater than 1.5, and copy number loss was defined as a ratio less than 0.66. The percentage of chromosomal regions showing copy number gain or loss relative to the sample's ploidy was calculated for each autosomal chromosome. Subsequently, the mean percentage value was computed as the weighted Genome Instability Index (wGII) score.

**Transcriptome data processing procedure**

Unwanted variations from non-biological biases were adjusted by the RUVSeq package (version 1.34.0) based on housekeeping genes obtained from the HRT Atlas database. The relative abundance of immune cell infiltration in the tumor microenvironment was estimated using xCell, and differences between SYEC2 and SYEC21 tumor samples were assessed using two-sided Wilcox tests.

Differential expression analysis was conducted using the DESeq2 R package (version 1.36.0), applying criteria of |fold change|>2 and a false discovery rate (FDR) of <0.05 to identify significant genes. These differentially expressed genes were further analyzed using Gene Set Enrichment Analysis (GSEA) from the clusterProfiler package, employing signatures from the MSigDB hallmark collection of mouse version, which encapsulates well-defined biological states or processes.

**Statistical analysis**

Statistical analysis was conducted using GraphPad Prism 8.0 (GraphPad Software, San Diego, California, USA). Differences between groups of figure 1 were evaluated using Two-way ANOVA (G-H) and One-way ANOVA (J-N), and supplementary figure 2 were evaluated using Student's t-test. A significance level of P < 0.05 was considered statistically significant, while P > 0.05 indicated no statistical significance. Statistical significance levels were denoted as follows: *, *P* < 0.05, **, *P* < 0.01, ***, *P* < 0.001, NS, not significant.
